# Supplementary material for: 100 GHz micrometer-compact broadband monolithic ITO Mach–Zehnder interferometer modulator enabling 3500 times higher packing density
Source: Nanophotonics. 2022 Apr 8;11(17):4001–9. doi: 10.1515/nanoph-2021-0796 (PMC11501683; doi:10.1515/nanoph-2021-0796)
Supplement: Supplementary file 1 — Supplementary Material [file j_nanoph-2021-0796_suppl_001.docx]

**100 GHz Micrometer-compact broadband Monolithic ITO Mach–Zehnder Interferometer Modulator enabling 3500 times higher Packing Density**

**Supplementary Information**

Yaliang Gui^1^, Behrouz Movahhed Nouri^1^, Mario Miscuglio^1^, Rubab Amin^1^, Hao Wang^1^, Jacob B. Khurgin^2^, Hamed Dalir^1^, and Volker J. Sorger^1^

^1^George Washington University, 800 22nd Street NW, Washington, DC 20052, USA

^2^Johns Hopkins University, Baltimore MD 21208, USA

**^+^**Corresponding authors: [hdalir@gwu.edu](mailto:hdalir@gwu.edu) [sorger@gwu.edu](mailto:sorger@gwu.edu)

**i. Performance analysis of arbitrary power splitter based MZM (Plasmonic mode)**

An asymmetric power splitter based on MZI consists of an input Y junction with a particular splitting ratio and an output Y junction with a 50:50 splitting ratio. On the active arm, a plasmonic mode ITO modulator is used to shift the phase. We will utilize a plasmonic mode ITO modulator as an example because to its advantages, which include a compact footprint, the possibility for high speed, and a large modulation depth. However, as Kramers–Kronig relations indicate, a high modulation depth is associated with a high propagating loss.

This article also discusses the effect of spreading loss. For π shift device, high ER can be achieved by compensating device propagating loss with higher splitting power (splitting ratio > 50) or using balance device on the passive arm. Low IL can be achieved at the same time by changing the device length or by using different MZI arm lengths. By using asymmetric arms, the initial phase difference, $\Delta\varphi_{initial}$ , caused by different effective mode index of two arms can be precisely controlled (fig a, b,8c). Depending on the input signal, the initial phase difference, $\Delta\varphi_{initial}$, is the same with the OFF state (0V) phase difference for applications that use either positive or negative bias, while for applications that use both positive and negative bias, OFF state phase difference under negative bias would be discussed instead of the initial phase difference. Since phase difference shift forward under negative bias and vice versa. The transmission ER and IL can be calculated by using:

|  | $T=\frac{{{(\frac{\sqrt{x}}{10}E}_{in}e^{-\frac{\alpha}{2}L})}^{2}+{{(\frac{\sqrt{100-x}}{10}E}_{in})}^{2}}{2}+\frac{2\times{(\frac{\sqrt{x}}{10}E}_{in}e^{-\frac{\alpha}{2}L})\times{(\frac{\sqrt{100-x}}{10}E}_{in})\times cos(\Delta\varphi_{initial}+\Delta\varphi_{bias})}{2}$ | (1) |
| --- | --- | --- |
|  | $ER=10\cdot lg(\frac{T_{\max}}{T_{\min}})$ | (2) |
|  | $IL=10\cdot\lg\left( T_{\max} \right)+L_{facet}\times2$ | (3) |
|  | $\Delta\varphi_{bias}= \pi$ (For π phase shift device) | (4) |

( $E_{in}$, $x$ ,$\alpha$, $L$, $\Delta\varphi_{initial}$, $\Delta\varphi_{bias}$ and $L_{facet}$ are the input electric field phasor, the splitting ratio on the active arm side, device absorption coefficient, device length, the initial phase difference caused by different effective mode index between device and waveguide, phase changed by bias and facet coupling loss). Here we assume there is a π phase shift ITO plasmonic device with effective mode index of 2.5, $\alpha\cdot L=0.2$ under 0 V and $\alpha\cdot L=0.26$ under $V_{\pi}$. (fig. 1e and 1f) The initial phase difference is eliminated by using different MZI arms or using balanced device. $L_{facet}$ is 0.07dB obtained from Lumerical simulation. Power with high splitting ratio (x=60) on the active arm compensate the propagating loss and is used in application requires high ER. Low splitting ratio power (x=37) in contrast decrease the insertion loss by reducing the modulation depth. The overall transmissions decrease with increasing power splitter ratio because of the increasing propagating losses. Symmetric MZI with balance device has as high ER as asymmetric MZI modulator (60:40) but has 8% weaker transmission. Device with free choice of power splitting ratio is preferred than the device with balance device considering the IL.

For small phase shift device, high ER and low IL cannot be obtained at the same time. The free choice of splitting ratio provides more options for different application. High splitting ratio for applications that requires high ER, while low splitting ratio for applications with high IL requirements. What’s more, the $\Delta\varphi_{initial}$ should be carefully chosen for different applications.


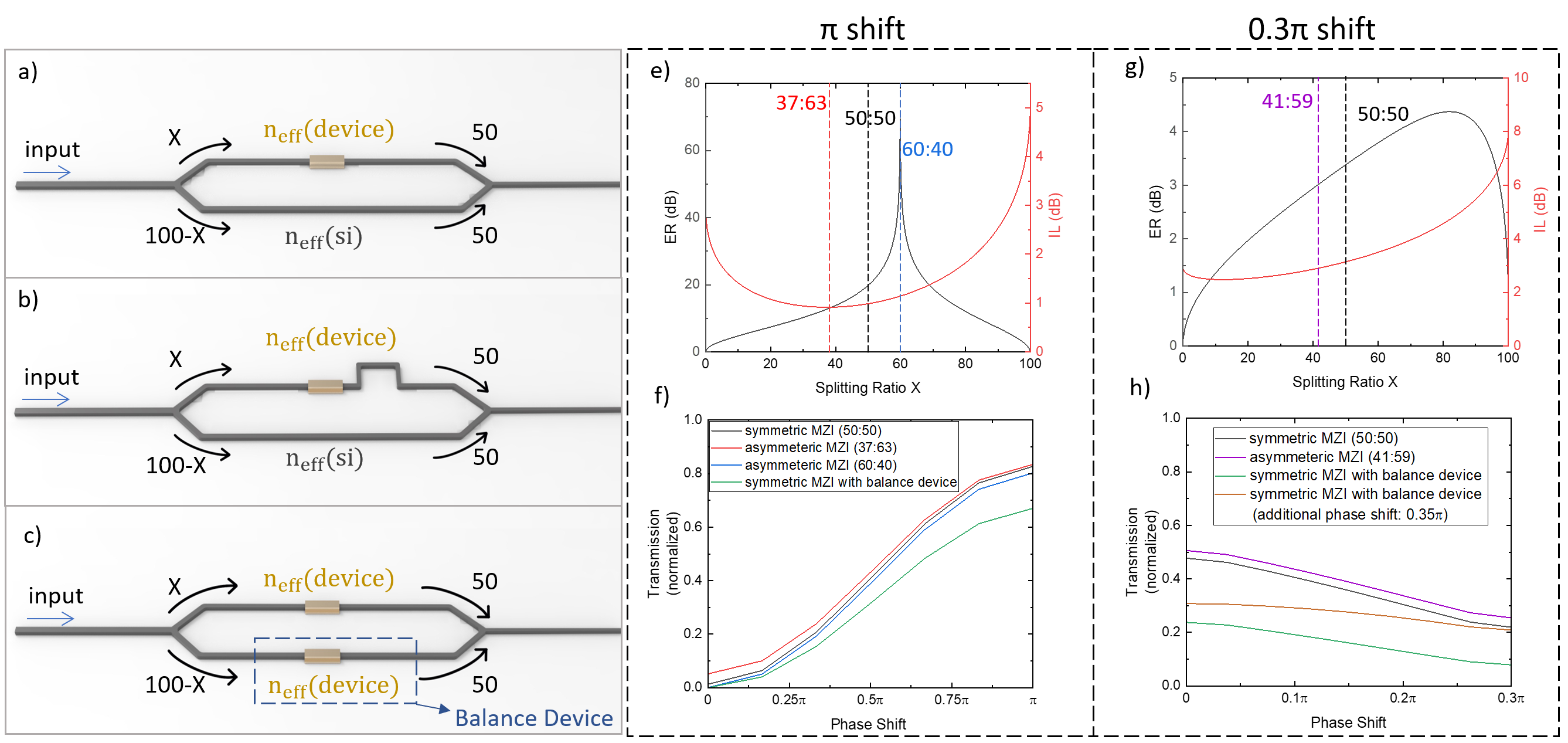


Figure 1 Performance analysis for MZI modulator with free choice power splitting ratio. a) MZI modulator with arms of the same length. b) MZI with different arm length which is used to tune the OFF-state phase shift to kπ, k=0,1,2…. c) MZI modulator with balance device on passive arm. The initial phase shift is 0. e) ER and IL of π phase shift device under different splitting ratio. (Device with power splitting ratio of 37:63 has lowest IL of 0.9dB and ER of 13dB; device with power splitting ratio of 50:50 has IL of 1dB and ER of 20dB, and device with power splitting ratio of 60:40 has IL of 1.1dB and ER of 70dB.) f) Normalized transmission of different splitting ratio (50:50, 37:63 and 60:40) and with balance device under bias with π phase shift. IL and ER of symmetric MZI modulator with balance device is 1.7dB and >70dB respectively. g) ER and IL of $0.3\pi$ phase shift device under different splitting ratio. h) Normalized transmission of different splitting ratio (50:50, 37:63 and 60:40) and with balance device under bias with $0.33\pi$ phase shift.

Here, plasmonic mode ITO-based MZI modulator $0.33\pi$ phase shift is used as an example of small phase shift device. The performances including the ER and IL is lower than previous π shift design because of weak modulation depth. However, it’s useful for most of applications. The performance of symmetric MZI modulator with balance device can be optimized according to the application by using additional phase shift. Still comparing with the asymmetric MZI modulator, it’s not a good choice.

**ii. Selection & Thickness of Dielectric Material**

**
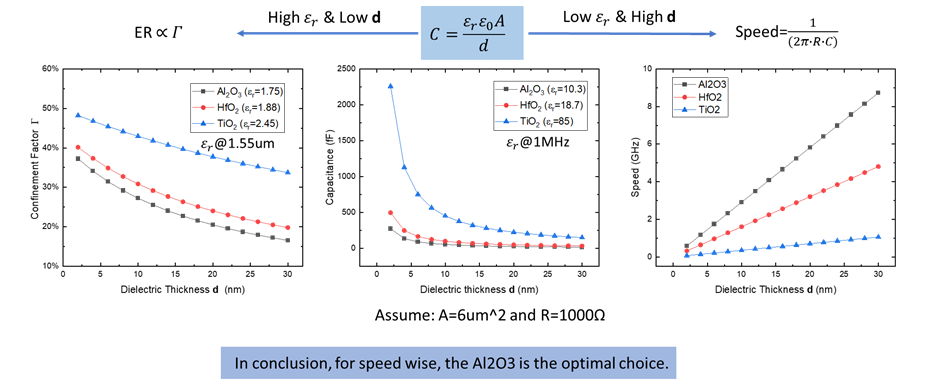
**

**iii. Photonic mode based on an arbitrary power splitter modulator ITO MZI**


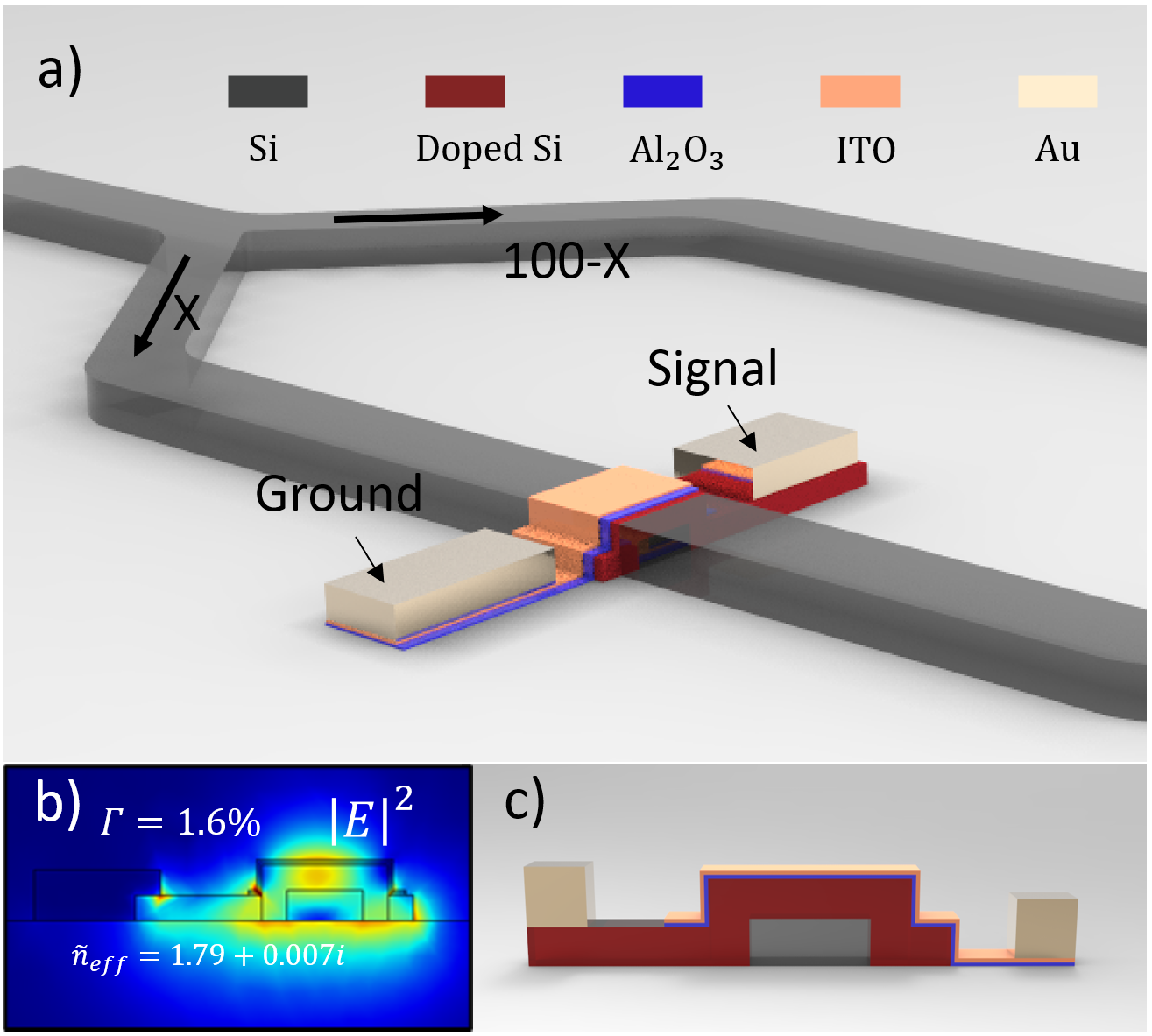


Figure 2 ITO-based Photonic mode MZI modulator on Si photonic platform. (a) Perspective view of the Mach-Zehnder structure with the active biasing contacts. The power splitting ratio for the active arm is X, and the splitting ratio for the passive arm is 100-X. (b) Electric field distribution by performing FEM at 1550nm under +3.5V in the cross-sectional structure for a z cutline along the central region of the Si waveguide (width: 500$\mathrm{nm}$; height: 220$\mathrm{nm}$). (c) Composition layers of ITO MZI modulator. ($T_{doped silicon}=100nm;$ $T_{{Al}_{2}O_{3}}=10nm;$ $T_{\mathrm{ITO}}=10nm;$ $T_{\mathrm{Au}}=50nm)$

**Photonic mode ITO based modulator design:** The photonic mode ITO-based modulator is built on a silicon-on-insulator (SOI) platform and has an asymmetrical MZI. On the input side, a Y-junction has been carefully designed as a power splitter with an arbitrary (non-50:50) splitting ratio determined by device loss and a 50:50 Y-junction on the output side to balance the power of each arm. For the photonic mode device option, silicon is doped at the device area as the bottom contact with a doping concentration of 1020 cm^-3^, resulting in a conductivity of 97087 S/m. The doping depth must not be too thin in order to maintain a low resistance and was chosen in this case to be 100nm. At 1550nm, the complex refractive index[1] of doped silicon is calculated as follows:

$$n_{doped}=n_{intrinsic}+\Delta n$$

$$k_{doped}=k_{intrinsic}+\Delta k$$

$$\Delta\alpha={(4\cdot\Delta k\cdot\pi)}/\lambda$$

$\Delta\alpha=\Delta\alpha_{e}+\Delta\alpha_{h}$=8.88$\times{10}^{-21}\times\Delta{N_{e}}^{1.167}$+5.84$\times{10}^{-20}\times\Delta{N_{h}}^{1.109}$

$-\Delta n=\Delta n_{e}+\Delta n_{h}$=5.4$\times{10}^{-22}\times\Delta{N_{e}}^{1.011}$+1.53$\times{10}^{-18}\times\Delta{N_{h}}^{0.838}$

Where, $\Delta\alpha_{e},$ $\Delta\alpha_{h}$,$\Delta n_{e}$, $\Delta n_{h},$are absorption coefficient change by doped electrons and holes, respectively, and the refractive index change again arising from doped electrons and holes, number of electrons introduced through doping, respectively. $\Delta N_{e} and \Delta N_{h}$ are the carrier concentrations for both charges introduced by doping. $n_{intrinsic}$=3.48 and $k_{intrinsic}=0 .$The carrier concentration of unbiased ITO thin film is $2.07\times{10}^{20}{cm}^{-3}.$ Under ±5V, the refractive indexes for 10nm ITO thin film are 1.7896+0.007686i and 1.6539+0.00261i. a 10nm thin Al_2_O_3_ film between ITO thin film and doped silicon region is used as gating oxide. The anticipated speed is 108GHz.


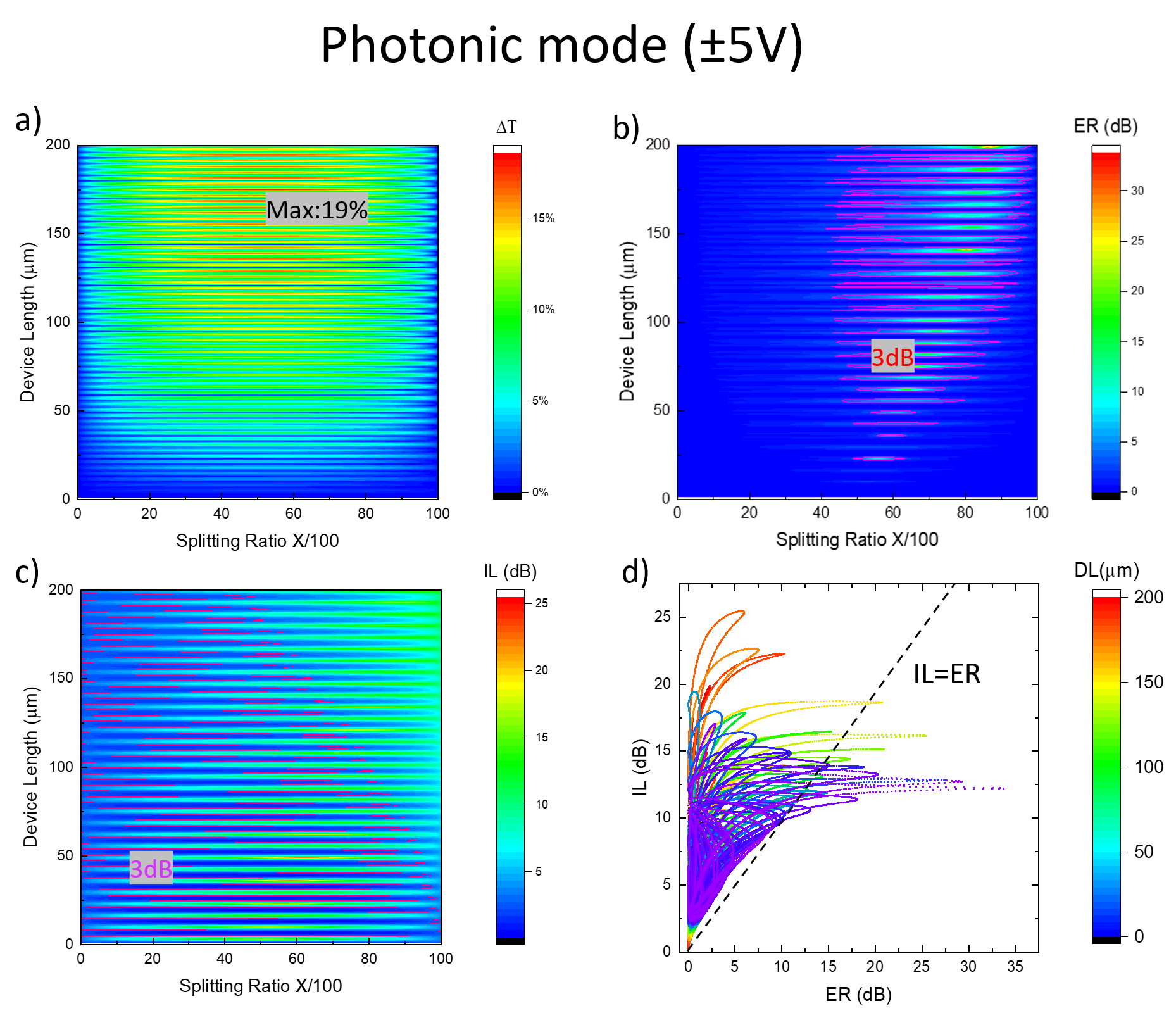


Figure 3 The performance map of asymmetric photonic mode ITO-based MZI modulator with 15nm ${Al}_{2}O_{3}$ under ON (-5V) and OFF states (+3.5V). a) Transmission difference varies with splitting ratio X and device length 0 𝜇𝑚 to the maximum transmission difference of the photonic mode device is 19% because of weak modulation depth. Photonic mode designs with high transmission differences locate in long-length regions because of weak modulation depth and low device loss. b) Extinction ratio of photonic device varies with splitting ratio for different lengths. c) Insertion loss of plasmonic mode and photonic mode varies with splitting ratio for different lengths. d) Performance map for device length ranging length from 0.1um to 20um.
